# Research on Friction Welded Connections of B500SP Reinforcement Bars with 1.4301 (AISI 304) and 1.4021 (AISI 420) Stainless Steel Bars

**DOI:** 10.3390/ma19020313

**Published:** 2026-01-13

**Authors:** Jarosław Michałek, Ryszard Krawczyk

**Affiliations:** 1 Faculty of Civil Engineering, Wrocław University of Science and Technology, Wybrzeże Stanisława Wyspiańskiego Street 27, 50-370 Wrocław, Poland; 2 Faculty of Mechanical Engineering, Częstochowa University of Technology, J.H. Dąbrowskiego Street 69, 42-201 Częstochowa, Poland; ryszard_krawczyk@wp.pl

**Keywords:** foundation pile, traction pole, friction welding, stainless steel, research

## Abstract

Steel and prestressed concrete traction poles can be fixed to reinforced concrete pile foundations using typical bolted connections. The stainless steel fastening screw is connected to the ordinary steel foundation pile reinforcement by friction welding under specific friction welding process parameters. From the perspective of the structural strength of the connection between the traction pole and the foundation pile, regarding the transfer of tensile and shear forces through a single anchor bolt, the yield strength of stainless steel bolts should be R_e,min_ ≥ 345 MPa for M30 anchors, R_e,min_ ≥ 310 MPa for M36 anchors and R_e,min_ ≥ 300 MPa for M42 anchors. This requirement is reliably met by martensitic stainless steels, while other stainless steels have yield strengths below the required minimum. What truly determines the foundation pile’s load capacity is not the satisfactory mechanical strength of the stainless steel (here, the parameters are met), but the quality of the friction-welded end connection between the reinforcement and the threaded bars. Incorrect selection of the type of prestressing steel in the analyzed connection can have enormous consequences for foundation pile manufacturers. Annual production of foundation piles amounts to thousands of units, and an incorrect decision made by the pile designer at the design stage can result in significant financial losses and a high risk to human life. This article presents the results of studies on friction-welded connections of M30, M36, and M42 threaded bars made of austenitic 1.4301 (AISI 304) and martensitic 1.4021 (AISI 420) stainless steel with B500SP reinforcement bars. The tests yielded negative results for 1.4021 (AISI 420) steel, despite its yield strength exceeding R_e_ ≥ 360 MPa.

## 1. Introduction

### 1.1. Pile Foundations for Railway Traction Poles

**Figure 1:**
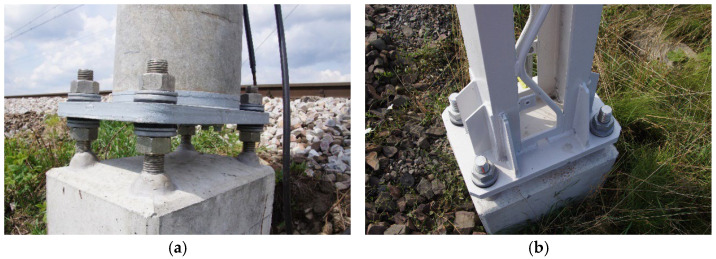
Connection of the foundation pile to the column made of: (**a**) prestressed concrete; (**b**) steel (photo by J. Michałek).

The fastening of railway traction support structures to foundations includes traditional methods (direct foundations) and modern methods (prefabricated pile foundations) [1,2]. In the traditional method, the support structure is placed in a trench (sometimes in a steel pipe or concrete rings), which is then concreted. Another solution for the direct foundation of traction poles is the use of monolithic or prefabricated cup foundations. In a more modern version, ready-made foundation piles (usually with a maximum length of 5.5 m [3]) are used, driven into the ground, to which the supporting structure (steel or pre-stressed concrete—Figure 1 [4,5]) is screwed.

Foundation piles of types P and B (letters identifying producers) are prefabricated reinforced concrete elements installed by driving them directly into the ground. Traction pile quality and reliability procedures require control during production, verification of their load-bearing capacity, and checking the pile’s structural continuity once in the ground [6]. Each pile foundation consists of two main parts: a wider head section that protrudes above the ground and a narrower shaft section that remains embedded in the soil. Traction piles are reinforced concrete foundations, sized in 0.5 m increments from 2.5 to 5.0 m, designed to secure steel or prestressed concrete traction poles (ETG from abbr. PL Elektroenergetyczny słup Trakcyjny z Głowicą stalową = Electric power traction pole with steel head) [5]. The connection is made with four stainless steel threaded anchors (M30, M36, or M42, see Figure 1); the specific size depends on the foundation class (I, II, or III) and the pole model. In addition to the traction piles, there are two types of anchor piles (I-K and III-K) without bolts, also 2.5–5.0 m long in 0.5 m increments; these are used for securing the stays of center poles and heavy anchor foundations.

**Figure 2:**
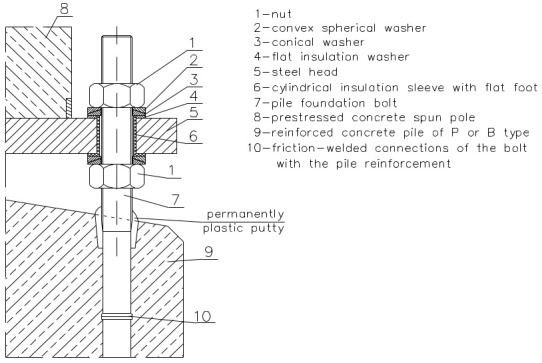
Detail of the connection of the prestressed concrete pole head with the foundation pile.

The foundation piles (types I, I-K, II, III, and III-K) are reinforced with #28 mm ribbed longitudinal bars made of B500SP steel [7]. This steel has a characteristic yield strength f_yk_ ≥ 500 MPa (E_s_ = 200 GPa, f_yk_ = 500 MPa, f_yd_ = 500/1.15 ≅ 435 MPa), which is assumed to meet all requirements. The four corner reinforcement bars are friction-welded to the threaded bars M30, M36, or M42 made of stainless steel. The steel traction pole head is electrically insulated from the M30, M36, and M42 threaded bars (and, consequently, from the pile reinforcement) using insulating washers and sleeves (Figure 2). This crucial step protects the pile reinforcement from electrolytic corrosion caused by stray currents.

The optimal solution for the design of a foundation pile would be for the #28 mm reinforcement bars to be continuous and to serve simultaneously as the anchoring element for the traction pole. However, environmental conditions and the PKP PLK S.A. (PKP Polish Railway Lines S.A.) instruction [8] require that the external section be made of stainless steel. Producing a foundation pile with fully stainless-steel reinforcement is not cost-effective. As a solution, the ordinary steel reinforcement (protected by concrete) is now friction-welded to a segment of stainless steel threaded rod (M30, M36, or M42). The aforementioned instruction [8], in addition to friction welding between the pile reinforcement and threaded anchors, also allows for butt welding.

Stainless steel is classified into five different “families” of alloys, each with a distinct set of characteristics [9]. Four of these families are defined by their dominant crystal structure—namely austenitic, ferritic, martensitic, and duplex alloys. The fifth family, precipitation-hardening steels, is defined by the type of heat treatment used to develop its properties [10].

For a connection between a traction pole and a foundation pile to maintain adequate structural strength and effectively transfer tensile and transverse forces via a single anchoring bolt, the stainless steel used for the bolts must meet specific minimum yield strengths: R_e,min_ ≥ 345 MPa for M30 anchors, R_e,min_ ≥ 310 MPa for M36 anchors and R_e,min_ ≥ 300 MPa for M42 anchors. This requirement is reliably met by martensitic stainless steels (Table 1). The remaining types of stainless steel have minimum yield strengths below the required values.

However, the critical point of the foundation piles determining their load-bearing capacity is not the mechanical strength parameters of the stainless steel (these are satisfactory), but the butt joint created by friction welding between the reinforcing bars of the pile and the threaded bars. From the point of view of the foundation pile design, it is sufficient to consider only the strength requirements of the steel friction connection, allowing the designer to confirm the load-bearing conditions in terms of the transfer of tensile and transverse forces through a single fastening anchor. However, the friction connection of steel in the foundation pile structure, especially when using martensitic steel 1.4021 (AISI 420) in combination with the adopted welding process (although this is not a welding problem), can lead to serious failure or disaster, which in turn can result in significant financial losses and a high risk to human life.

This paper presents the results of tests on friction-welded joints connecting threaded bars M30, M36, and M42 made of austenitic stainless steel 1.4301 (AISI 304) and martensitic stainless steel 1.4021 (AISI 420) with reinforcing bars made of B500SP steel. This demonstrates how important it is to fully evaluate the friction connections of bars. Simplified tensile strength tests on unmachined samples were performed at the Structural Engineering Laboratory (Department of Building Structures, Faculty of Civil Engineering, Wrocław University of Science and Technology). The main, comprehensive testing of the friction-welded joints took place at the Technical Control Laboratory of PIK SPAW S.C. (proper name) in Częstochowa (Poland) [13].

### 1.2. Friction Welding

**Figure 3:**
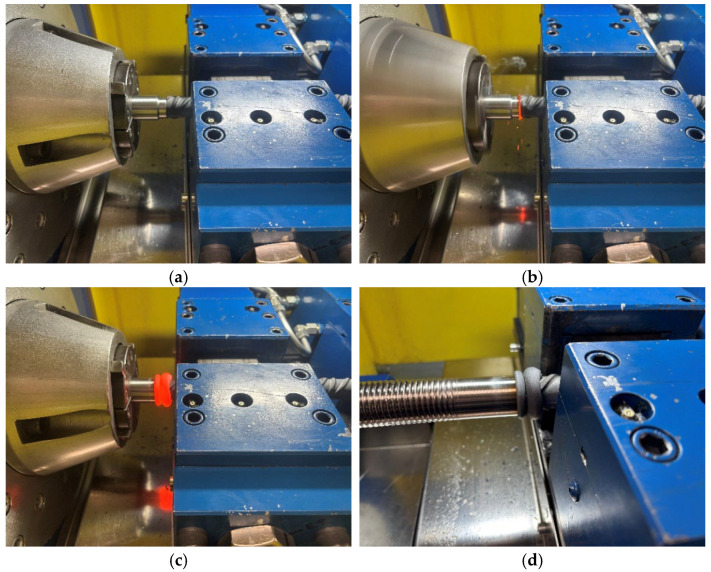
Stages of friction welding: (**a**) two materials pressed together, (**b**) friction phase—the contact surfaces heat up due to rotation and applied pressure, (**c**) upsetting phase—the highly plasticized metal is extruded radially outward from the joint, forming a weld flash, (**d**) forging and cooling phase—both materials cool down together (photo by M. Siwiec).

Rotary Friction Welding (RFW) joins materials by generating heat through friction in the contact area. This direct conversion of mechanical energy to thermal energy plasticizes the materials, allowing them to bond [14,15,16]. The subsequent rotation of one material around the joint axis defines this initial stage as the friction phase. The contact surfaces heat up due to relative motion (rotation) and the applied pressure (Figure 3a). The process of friction and heat generation (the friction phase—Figure 3b) continues until both materials reach the required welding temperature in the joint area, which is usually below the melting point of the metals being joined. Friction welding is frequently referred to as solid-state welding because the materials never fully melt. Following the friction stage, the relative motion is halted, and an upsetting force (forging pressure) is applied to form a durable metallic bond; this is the upsetting phase (Figure 3c). The applied upsetting force causes the highly plasticized metal to be extruded radially outward from the interface, forming a flash around the weld, while the two welded materials gradually cool down together, allowing the structure in the joint area to crystallize (the forging and cooling phase—Figure 3d).

Numerous studies [16,17,18,19,20,21,22,23,24,25] have investigated the influence of various parameters on the properties of friction-welded joints, such as friction pressure and time, upset pressure and time, rotational speed, oscillation frequency, oscillation amplitude, and friction pressure, as well as their correlation with microstructural characteristics, microhardness fluctuations, interphase formation, optimal welding parameters, and mechanical properties. The selection of friction welding parameters is critical as it dictates the heat generation and material flow during the friction welding process, which in turn controls the resulting weld’s microstructure, residual stresses, and structural integrity. The influence of each friction welding parameter largely depends on the type of materials being joined, including both similar and dissimilar combinations.

## 2. Materials and Research Methods

The article presents the results of research on friction-welded connections of foundation pile reinforcing bars with stainless steel threaded bars used as anchors for fixing railway traction poles. For the tests in question, #28 mm ribbed bars made of B500SP steel [7] were used in the production of pile foundations. M30, M36, and M42 threaded bars are made of austenitic stainless steel grade 1.4301 (AISI 304) and martensitic stainless steel grade 1.4021 (AISI 420). Reinforcing bars and threaded bars were joined together by friction welding. M30, M36, and M42 threaded bars in the welding area were previously machined to the diameter of the reinforcing rod, i.e., #28 mm over a length of up to 35 mm. The friction welding parameters for threaded bars with reinforcing bars are shown in Table 2. The tests were carried out in accordance with the requirements of standard [26] for each batch, taking into account the diameter of the threaded rods and the two types of materials used: 1.4301 (AISI 304) and 1.4021 (AISI 420). The following tests were performed: visual assessment of all samples from each batch, tensile and flexural tests for two samples, impact tests for three samples, and macroscopic and hardness distribution tests for one sample.

To confirm that the minimum yield strength of austenitic stainless steel grade 1.4301 (AISI 304) exceeds R_e_ = 345 MPa, basic tests were carried out on M30, M36, and M42 threaded bars. The tests were conducted at the Metallography and Heat Treatment Laboratory in Mikołów (Poland) in accordance with the standard [27]. Two samples of each diameter were tested using LabTest 6.250 and ZD100PU class 1 testing machines. The samples were not aged prior to testing. All tests were conducted at a temperature of approximately +20 °C. The tests were conducted to measure the following mechanical properties: tensile strength (R_m_), yield strength (R_e_), and elongation at break (A_5_). All samples were machined prior to testing.

Visual inspections of friction-welded joints of M30, M36, and M42 threaded bars made of austenitic stainless steel 1.4301 (AISI 304) and martensitic stainless steel 1.4021 (AISI 420) with #28 mm B500SP reinforcing bars were performed in accordance with standard [28] at the Technical Control Laboratory in Częstochowa (Poland) [13]. All samples delivered for testing were examined. As a fundamental quality assessment method for welded joints, visual inspection is crucial because it facilitates the early detection of potential discontinuities. These issues include cracks, undercuts, weld geometry problems, and flash irregularities. Inspections were conducted from a distance of no more than 600 mm from the surface, with a minimum observation angle of 30 degrees, using a caliper, weld gauge, 5× magnifying glass, and a light source with intensity above 500 Lx.

**Figure 4:**
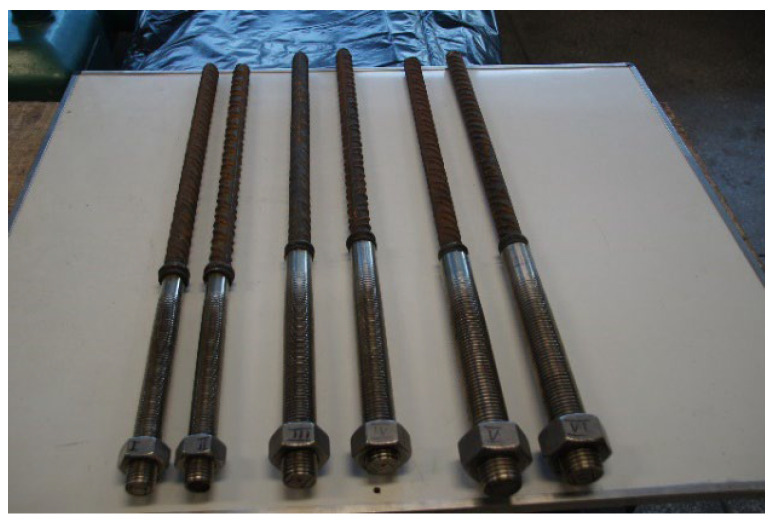
Samples for testing the effectiveness of the butt joint of a #28 mm reinforcing bar with a stainless steel bolt (from left: M30, M36, M42) made of 1.4301 (AISI 304) stainless steel (photo by J. Michałek).

Simplified tensile tests were carried out at the Structural Engineering Laboratory of the Department of Building Structures, Faculty of Civil Engineering, Wrocław University of Science and Technology (Poland). Tests of butt joints between #28 mm reinforcing bars with a characteristic yield strength f_yk_ ≥ 500 MPa (B500SP steel) and friction-welded stainless steel fastening bolts (M30, M36, and M42 of austenitic stainless steel 1.4301 (AISI 304)) connecting the pole to the foundation pile were performed according to standard [29] on two samples for each foundation pile type PI, PII, and PIII. The tensile tests on the butt joints were performed on unmachined samples (Figure 4). A ZD100 testing machine (class 1) with a maximum capacity of 1000 kN was used for the procedure. The samples were not subjected to any aging and the tests were carried out at a temperature of +21 °C.

**Figure 5:**
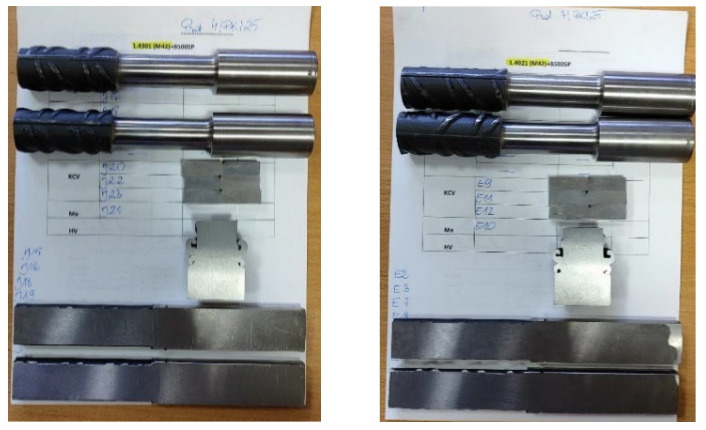
Sample sets for testing (from top): tensile strength, impact toughness, metallographic analysis, and bending strength, shown for a butt joint of a #28 mm reinforcing bar with an M42 bolt made of stainless steel 1.4301 (AISI 304)—left and 1.4021 (AISI 420) (photo by R. Krawczyk).

Tensile strength tests of the butt joints between #28 mm reinforcing bars and fastening bolts (M30, M36, and M42 made of austenitic stainless steel 1.4301 (AISI 304) and martensitic stainless steel 1.4021 (AISI 420)) were carried out using a Mohr & Federhaff AB class 1 testing machine with a maximum load capacity of 350 kN [14]. The tests were performed at ambient temperature (+20 °C), adhering to standard [30]. For each diameter of threaded rod and stainless steel grade, two machined samples (Figure 5) were used. The measurement length of the test specimens covered the entire friction-welded joint zone.

Transverse bending tests of the friction-welded joints of #28 mm reinforcing bars with bolts (M30, M36, and M42 of 1.4301 (AISI 304) and 1.4021 (AISI 420) stainless steel) were performed on the same Mohr & Federhaff AB class 1 testing machine, also at +20 °C, according to standard [30], with two samples for each rod diameter and stainless steel grade. After being taken transversely from the weld, all bending samples were machined so the weld axis remained cantered. The final dimensions were a thickness of 12.5 mm and a width of 26 to 31 mm (equal to the minimum thickness of the material next to the joint).

During the bending test, the sample was placed on two parallel rollers spaced 80 mm apart, with the weld axis positioned at the midpoint between the rollers. Using a 50 mm diameter pin, the sample was loaded in three-point bending (at the centre of the span, perpendicular to the surface, and along the weld axis). The test was completed when the bending angle exceeded 90°.

Impact toughness tests of the friction-welded butt joints of #28 mm reinforcing bars with fastening bolts (M30, M36, and M42 made of austenitic stainless steel 1.4301 (AISI 304) and martensitic 1.4021 (AISI 420)) were performed using a Charpy PW-300 impact hammer with an initial energy of 300 J [13]. The samples were cut from the welded joint with their longitudinal axes perpendicular to the weld length. The reference line for the sample was set to align with the centreline of the weld. A notch (45° angle, 2 mm height) was made transversely to the sample’s main axis in the centre of the sample (designated VWT), with the notch centre aligned with the reference line (a = 0). The distance from the welded joint face to the nearest sample surface was b = 9 mm. The final sample designation, indicating type, notch position, orientation, and distance from the reference line, was VWT 0/9. The cross-sectional dimensions of the full-size impact sample were 10 × 10 mm, with a section of 10 × 8 mm at the notch (cross-sectional area under the “V” notch S = 80 mm^2^). Following standard [31], the tests were performed at ambient temperature (+20 °C). For every specific threaded rod diameter and stainless steel grade, three samples (Figure 5) were used.

**Figure 6:**
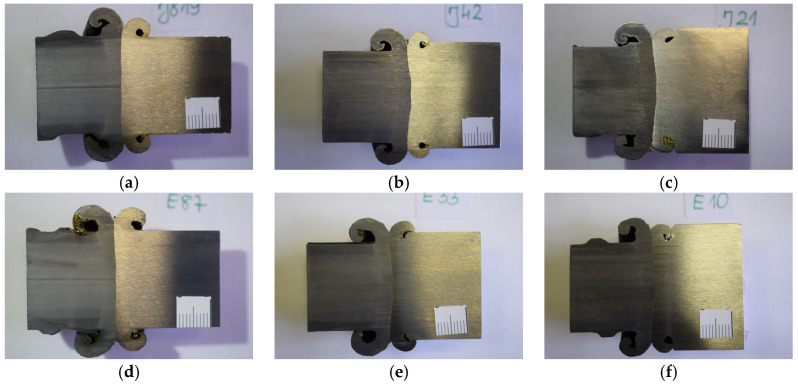
Macroscopic cross-sections of the connection between a #28 mm-B500SP reinforcing bar (left) and a threaded bar (photo by R. Krawczyk): (**a**) M30-1.4301 (AISI 304), (**b**) M36-1.4301 (AISI 304), (**c**) M42-1.4301 (AISI 304), (**d**) M30-1.4021 (AISI 420), (**e**) M36-1.4021 (AISI 420), (**f**) M42-1.4021 (AISI 420).

Macroscopic metallographic examinations of the friction-welded joints of M30, M36, and M42 threaded bars made of austenitic stainless steel 1.4301 (AISI 304) and martensitic stainless steel 1.4021 (AISI 420) with #28 mm B500SP reinforcing bars were conducted [13] both with the naked eye and using a magnifying glass under lighting up to 620 Lx, in accordance with standard [32]. A total of six samples—one for each rod diameter and stainless steel grade—were prepared. These samples were cut transversely across the joint to fully capture the weld, heat-affected zone, and base material (Figure 6). They were then ground and chemically etched using Nital and Villella reagents to expose the weld’s microstructure. All testing was conducted at ambient temperature (+21 °C).

Hardness measurements are a widely used method for evaluating the properties of steel materials. Hardness values allow assessment of material plasticity, which is particularly important for evaluating hardness distribution in various zones of welded or friction-welded joints. Higher hardness values in a weld compared to the base material frequently signal microstructural changes resulting from the heat generated during the welding or friction welding process.

**Figure 7:**
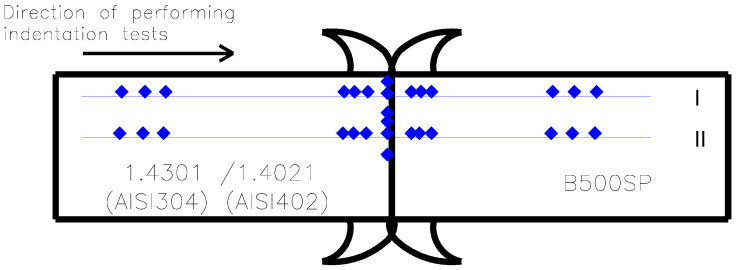
Scheme of material hardness testing along measurement lines I and II. Blue dots represent testing locations.

Hardness distribution tests of friction-welded butt joints of #28 mm reinforcing bars with fastening bolts (M30, M36, and M42 of austenitic stainless steel 1.4301 (AISI 304) and martensitic 1.4021 (AISI 420)) were conducted using a Vickers hardness tester HPO 10 [11] in accordance with standard [33]. The tester was verified using reference blocks (162.0 HV10 and 300.6 HV10). Samples were prepared by longitudinally cutting segments from the #28 mm butt joints that connected M30, M36, and M42 bolts. Hardness was then measured on the etched, transverse cross-section of the joint. Measurements followed two lines (I and II—Figure 7) at predetermined intervals to assess the impact of heat on hardness values. The first measurement line (I) was located 2 mm from the sample edge, while the second line (II) ran along the transverse axis of the welded joint (at the weld interface). The hardness tests were conducted at ambient temperature (+22 °C) using a 100 N indenter force. The investigation began along the measurement lines starting from the fastener bolt material, proceeding from the base material (BM_1_), through the heat-affected zone (SWC_1_), the weld itself, then again through the heat-affected zone (SWC_2_), to the base material (BM_2_) of the rebar (Figure 7).

## 3. Results and Discussion

Based on the basic tests of M30, M36, and M42 threaded bars made of austenitic stainless steel 1.4301 (AISI 304), it was determined that the nominal yield strength exceeds R_e_ = 360 MPa, which is higher than the values reported in the literature (Table 1). As detailed in Table 3, the results confirm that the fundamental strength criterion for the stainless steel (that the yield strength (R_e_) is greater than or equal to the minimum required yield strength (R_e_ ≥ R_e,min_) was successfully met for the traction pole-to-foundation pile connection.

A total of 60 friction-welded joint samples (10 for each rod diameter/steel grade combination: M30, M36, M42 bars in 1.4301 (AISI 304) austenitic and 1.4021 (AISI 420) martensitic stainless steel) were visually inspected. The results confirmed proper geometry and flash formation, with no defects found. All tested joints achieved the highest quality level B as defined by the stringent requirements of standard [34].

Results from the simplified tensile tests performed by the Structural Engineering Laboratory at Wrocław University of Science and Technology demonstrated that the butt joints friction-welded between #28 mm reinforcing bars and 1.4301 (AISI 304) stainless steel bolts (M30, M36, and M42) were properly executed. Samples of #28 mm bars joined with M30 threaded bars failed within the M30 bar material (R_m_ = 614 and 645 MPa), while samples joined with M36 bars (R_m_ = 649 and 650 MPa) and M42 bars (R_m_ = 650 and 654 MPa) failed within the reinforcing bar material. The measured tensile strengths (R_m_) align with the known tensile properties of both 1.4301 (AISI 304) stainless steel (Table 1) and B500SP steel [7]. Crucially, the friction-welded butt joints themselves remained intact and did not fail in any of the analysed cases.

**Figure 8:**
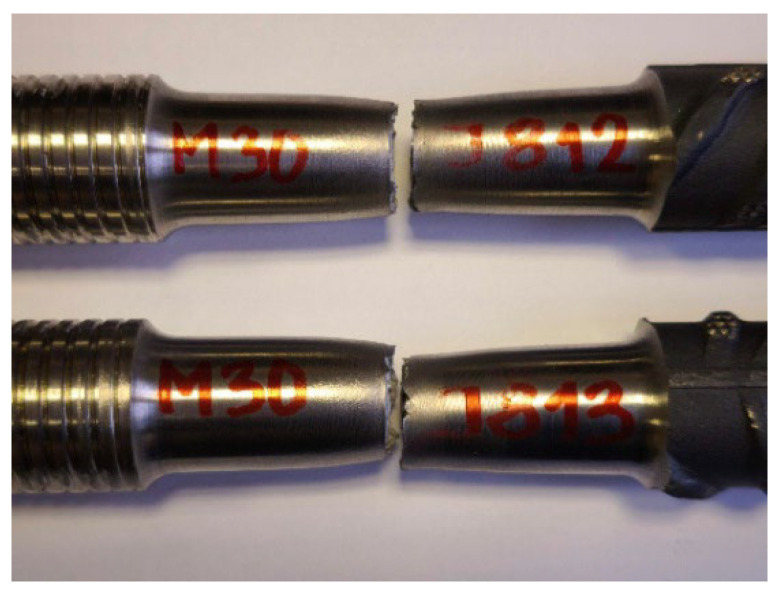
Samples of the #28+M30 connection after tensile testing; failure occurred in the weld zone (photo by R. Krawczyk).

**Figure 9:**
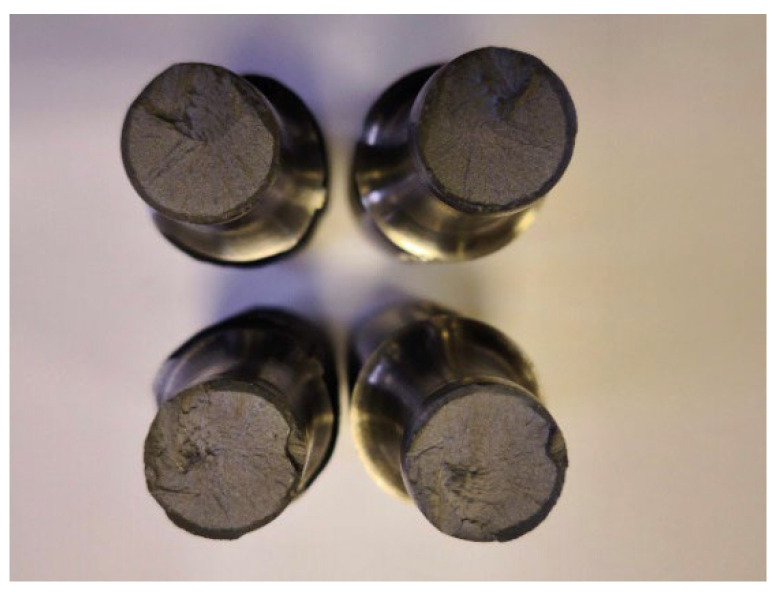
Fracture appearance of #28+M30 samples after tensile testing (photo by R. Krawczyk).

More detailed results from tests on machined samples [13] are presented in Table 4. All test results were positive, with failures occurring within the friction-welded joint zone (Figure 8). The tensile strengths achieved were similar for both stainless steel grades, and the fracture surfaces showed no defects (Figure 9).

**Figure 10:**
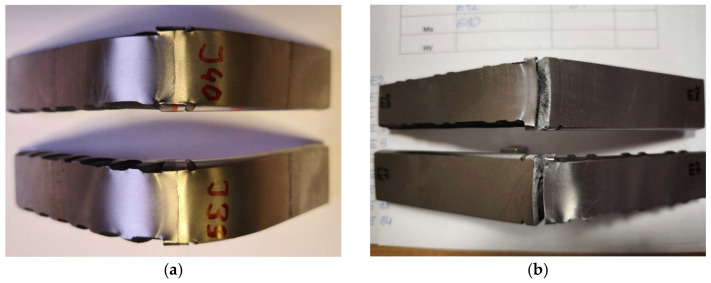
Samples after bending tests (photo: R. Krawczyk): (**a**) #28 + M36 samples without cracks after bending to 90°, (**b**) #28 + M42 samples with cracks over 3 mm wide at the weld zone at bending angles of 20° (lower sample) and 30° (upper sample).

The bending test results of friction-welded joints between M30, M36, and M42 threaded bars made of 1.4301 (AISI 304) stainless steel and #28 mm reinforcing bars confirmed the requirements of standard [26]. Each tested sample bent to a minimum angle of 90° without any cracking (Figure 10a). In contrast, the friction-welded joint samples combining 1.4021 (AISI 420) stainless steel (M30, M36, and M42 threaded bars) with #28 mm reinforcing bars performed differently. During the initial bending phase, at small deflections of approximately 15° to 30° (specifically, #28+M30 at 15° and 20°; #28+M36 at 18° and 20°; #28+M42 at 20° and 30°), cracks developed in the weld zone with an opening wider than 3 mm (Figure 10b). Consequently, these tests yielded a negative outcome and failed to meet the requirements of standard [26].

**Figure 11:**
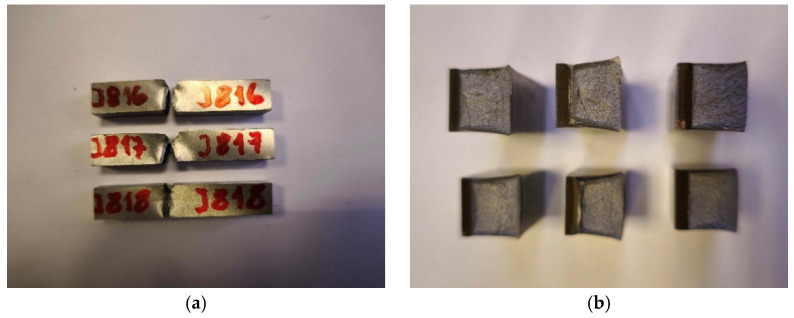
#28+M30 joint samples: (**a**) after Charpy hammer testing, (**b**) fracture surfaces (photo by R. Krawczyk).

Following standard [31], the notched bending impact test was carried out using a three-point bending setup in a Charpy impact hammer. The sample was positioned so the notch was precisely aligned with the pendulum’s strike point. The pendulum fractured the specimen with a single blow of specified energy. Analysis of the fracture surfaces showed a predominantly brittle character for all samples (Figure 11a).

Upon impacting the specimen, the pendulum releases part of its kinetic energy and therefore does not rise back to its initial drop height. The measured difference in height was used to determine the amount of absorbed energy KV, which represents the amount of energy consumed to achieve fracture of the tested element. The impact strength KCV was determined as the quotient of the fracture work KV and the cross-sectional area of the specimen under the notch S = 80 mm^2^. The average impact strength KCV for friction-welded joints of 28 mm diameter B500SP steel rebar with M30, M36, and M42 threaded bars made of austenitic stainless steel grade 1.4301 (AISI 304) was, respectively, KCV = 67.5, 44.6 and 35.3 J/cm^2^. For the martensitic steel 1.4021 (AISI 420), the following results were obtained, respectively: KCV = 37.9, 44.8, and 42.9 J/cm^2^. No defects were observed on the fracture surfaces of any of the samples (Figure 11b). All tested joint samples were deemed to have passed the impact test successfully.

Macroscopic metallographic examinations of the friction-welded joint samples of M30, M36, and M42 threaded bars made of austenitic stainless steel 1.4301 (AISI 304) and martensitic steel 1.4021 (AISI 420) with #28 mm reinforcing bars made of B500SP steel showed that the fusion line was correct. The flash shape was correct on both materials, but the volume of flash was larger on the reinforcing bar side. This suggests that the reinforcing bar experienced greater material shortening during welding. This larger flash volume may be attributed to the lower thermal conductivity of the stainless steel (due to its Cr content) compared to the B500SP steel [35]. Importantly, no internal defects were visible in the weld area.

Vickers hardness tests of friction-welded joints of M30, M36, and M42 threaded bars of stainless steel 1.4301 (AISI 304) and 1.4021 (AISI 420) with #28 mm B500SP reinforcing bars were conducted on a single sample for each rod diameter and steel type, giving a total of six samples. In the Vickers hardness test, the diagonals of the indentation left by the diamond indenter, shaped like a pyramid with a square base and a face angle of α = 136°, were measured optically. The Vickers hardness HV was calculated based on the average length of the measured diagonals d and the applied test force F = 100 N using the formula: HV = 0.1891 × F/d^2^. The obtained HV hardness distribution results for the parent material of the stainless steel bolt (MR_1_) and the reinforcing bar (MR_2_), heat-affected zones (SWC_1_ and SWC_2_), and the weld itself are shown in Table 5 and Table 6.

Tests confirmed that the 1.4301 (AISI 304) stainless steel connections to the B500SP reinforcing bar did not exceed the maximum allowable HV hardness values in the heat-affected zones and weld nugget (Table 5). For connections between martensitic stainless steel 1.4021 (AISI 420) and the B500SP reinforcing bar, the hardness distribution tests revealed exceedances of the permissible values in all types of connections. The hardness exceedances were observed in the heat-affected zone on the side of the 1.4021 steel and in the weld nugget itself—Table 6 [36]. Moreover, the higher hardness values in the weld zone were likely due to excessive heat generated during the friction welding process, resulting in too rapid cooling after completion [37]. The failed connections of the B500SP reinforcing bars and 1.4021 (AISI 420) steel threaded bars are attributed to a low level of material plasticity and the resulting brittle microstructures in the friction weld zone.

For single-bolt anchoring of the traction pole structure to the foundation pile, the stainless steel bolt yield strength must meet the following minimum requirements for transferring tensile and shear forces: R_e,min_ ≥ 345 MPa for M30 anchors, R_e,min_ ≥ 310 MPa for M36 anchors, and R_e,min_ ≥ 300 MPa for M42 anchors. This requirement is reliably met by martensitic stainless steels (Table 1). Other stainless steels have minimum yield strength values below the required threshold.

## 4. Conclusions

The article presents the problem of selecting the right type of stainless steel for the bolt used to attach the traction pole to the foundation pile. The stainless steel fastening screw is connected to the ordinary steel foundation pile reinforcement by friction welding under specific friction welding process parameters. Incorrect selection of the type of prestressing steel in the analyzed connection can be of great importance for pile manufacturers. The annual production of foundation piles amounts to thousands of units, and an incorrect decision made by the pile designer at the design stage may result in significant financial losses and a high risk to human life. This is particularly important because the designer relies mainly on the strength parameters of the connection between the traction pole structure and the foundation pile in terms of the transfer of tensile and transverse forces through a single fastening bolt. The article presents the results of tests on friction connections of M30, M36, and M42 threaded bars made of austenitic steel 1.4301 (AISI304) and martensitic steel 1.4021 (AISI420) with B500SP steel reinforcing bars. This demonstrates how important it is to fully evaluate the friction connections of rods, rather than just assessing their tensile strength.

Martensitic stainless steel grade 1.4021 (AISI 420) reliably meets the yield strength requirements for single anchoring bars used to transfer tensile and shear forces between the traction pole structure and foundation pile, specifically: R_e,min_ ≥ 345 MPa for M30, R_e,min_ ≥ 310 MPa for M36, and R_e,min_ ≥ 300 MPa for M42 anchors. Other stainless steels, including austenitic steel grade 1.4301 (AISI 304), have declared minimum yield strength values below the required threshold. Preliminary strength tests established that the nominal yield strength of austenitic steel grade 1.4301 (AISI 304) exceeds R_e_ = 360 MPa, thereby satisfying the minimum required yield strength (R_e_ ≥ R_e,min_) for stainless steel.

Based on these results, both stainless steel grades 1.4021 (AISI 420) and 1.4301 (AISI 304) are considered suitable for making the connection between the traction pole and the foundation pile. However, additional tests of friction-welded connections of M30, M36, and M42 threaded bars made of austenitic steel 1.4301 (AISI 304) and martensitic steel 1.4021 (AISI 420) with #28 mm B500SP reinforcing bars demonstrated that this assumption is incorrect.

Comprehensive testing—including visual and macroscopic evaluation, tensile, bending, impact, and hardness distribution tests—yielded fully positive results for the 1.4301 (AISI 304) steel connections. As an easily weldable austenitic stainless steel, the 1.4301’s (AISI 304) suitability for friction-welded connections was definitively confirmed by these results.

Tests conducted on friction-welded joints using 1.4021 (AISI 420) steel yielded positive results only in tensile and impact tests, while negative results were obtained in bending and hardness distribution tests. Both negatively evaluated tests showed the formation of hard structures in the joints, reducing plastic properties. Impact tests for these connections also showed significantly lower parameters by 20% compared to connections made of 1.4301 (AISI304) steel with B500SP steel reinforcing bars. Martensitic stainless steel 1.4021 (AISI 420) belongs to the group of difficult-to-weld, and sometimes unweldable, steels, which was confirmed by the negative test results. Negative test results do not allow for positive qualification of the welding technology for this series of bar joints according to standard [26].

As a result of the research conducted, it should be emphasized that in both cases, we are dealing with a category of hybrid or otherwise diverse connections, which in welded or heat-sealed connections cause greater problems than homogeneous connections. In the case described, significantly greater problems occurred when combining 1.4021 (AISI 420) steel with B500SP steel reinforcing bars.

In the future, additional research should be conducted based on other (as opposed to those shown in Table 2) technological parameters of the friction welding process in order to reduce the brittleness of the material in the area of the weld being formed. Recovery of plastic properties in the joint area will allow for positive bending and hardness results. If these conditions cannot be met, the alternative is to change materials within the same group to a steel with more favourable weldability, thereby eliminating the existing issues.

## Figures and Tables

**Table 1:** Mechanical properties of selected stainless steel grades.

Steel Grade	Yield Strength R_p0.2_ [MPa]	Tensile Strength R_m_ [MPa]	Elongation at Break A_5_ [%]
Austenitic stainless steel			
1.4305 (AISI 303)	min. 190 [11,12]	520–700 [11], 500–750 [12]	min. 35 [11,12]
1.4301 (AISI 304)	min. 230 [11], min. 190 [12]	540–750 [11], 460–700 [12]	min. 45 [11,12]
1.4307 (AISI 304L)	min. 220 [11], min. 190 [12]	520–670 [11], 460–700 [12]	min. 45 [11,12]
1.4541 (AISI 321)	min. 220 [11], min. 190 [12]	520–720 [11], 500–700 [12]	min. 40 [11,12]
1.4550 (AISI 347)	min. 220 [11], min. 205 [12]	520–720 [11], 510–740 [12]	min. 40 [11,12]
1.4401 (AISI 316)	min. 240 [11], min. 200 [12]	530–680 [11], 500–830 [12]	min. 40 [11], min. 30 [12]
1.4571 (AISI 316Ti)	min. 240 [11], min. 200 [12]	540–690 [11], 500–950 [12]	min. 40 [11], min. 25 [12]
Ferritic stainless steel			
1.4016 (AISI 430)	min. 280 [11], min. 240 [12]	450–600 [11], 400–630 [12]	min. 20 [11,12]
Martensitic stainless steel			
1.4021 (AISI 420)	min. 345 [11], min. 500 [12]	<700 [11], 700–1000 [12]	min. 15 [11],min. 8 [12]
1.4028 (AISI 420)	min. 345 [11], min. 600 [12]	<740 [11], 800–1000 [12]	min. 15 [11], min. 10 [12]
1.4031 (AISI 420)	min. 345 [11], min. 650 [12]	<760 [11], 850–1150 [12]	min. 12 [11], min. 7 [12]

**Table 2:** The friction welding parameters for threaded bars with reinforcing bars.

Welded Bars	Rotational Speed	Friction Phase		Pressure Phase		Shortening of Elements
		Pressure Force	Friction Time	Pressure Force	Friction Time	
	rpm	kN	s	kN	s	mm
#28+M30	800	65	11	110	6.5	15
#28+M36						
#28+M42						

**Table 3:** Tensile test results of M30, M36, and M42 threaded bars made of stainless steel grade 1.4301 (AISI 304).

Thread Diameter	Initial Cross-Section Area	Force Corresponding to 0.2% Elongation	Nominal Yield Strength	Maximum Tensile Force	Tensile Strength	Elongation at Break
	S_o_ [mm^2^]	F_p0.2_ [N]	R_p0.2_ [MPa]	F_m_ [N]	R_m_ [MPa]	A_5_ [%]
M30	309.15	131,083	424	218,063	705	47.33
	311.96	123,393	396	218,136	699	50.24
M36	416.44	150,000	360	295,500	710	50.34
	417.53	155,000	372	288,000	692	52.12
M42	705.76	220,000	527	470,000	1126	48.90
	694.50	270,000	647	455,000	1078	39.47

**Table 4:** Tensile test results of #28 mm reinforcing bars joined with M30, M36, and M42 threaded bars made of stainless steel grades 1.4301 (AISI 304) and 1.4021 (AISI 420).

Stainless Steel	Connection	Diameter After Machining	Initial Cross-Section Area	Maximum Tensile Force	Tensile Strength	Location of Break
		d [mm]	S_o_ [mm^2^]	F_m_ [N]	R_m_ [MPa]	
1.4301	#28+M30	22.0	380.0	237,000	624	weld nugget
		22.0	380.0	238,500	628	weld nugget
	#28+M36	22.0	380.0	240,000	632	weld nugget
		22.0	380.0	241,500	636	weld nugget
	#28+M42	21.9	376.5	238,000	632	weld nugget
		21.9	376.5	238,000	632	weld nugget
1.4021	#28+M30	22.0	380.0	248,000	653	weld nugget
		22.0	380.0	246,000	647	weld nugget
	#28+M36	22.0	380.0	237,000	624	weld nugget
		22.0	380.0	248,000	653	weld nugget
	#28+M42	21.9	376.5	245,000	651	weld nugget
		21.9	376.5	235,000	624	weld nugget

**Table 5:** Hardness test results of #28 mm reinforcing bar joints with M30, M36, and M42 threaded bars made of steel 1.4301 (AISI 304).

Stainless Steel	Joint	Measurement Line	Hardness HV				
			MR_1_	SWC_1_	Welded Zone	SWC_2_	MR_2_
1.4301 (AISI 304)	#28+M30	I	287	245	231	201	206
			285	230	228	202	203
			285	240	226	198	180
		II	285	274	220	205	168
			293	222	225	202	180
			280	230	228	200	170
	#28+M36	I	290	245	232	201	170
			285	248	229	203	172
			317	230	227	205	169
		II	285	287	249	206	187
			240	268	228	203	180
			297	264	232	200	173
	#28+M42	I	231	289	283	220	260
			221	288	245	233	288
			257	272	254	214	283
		II	225	230	217	173	182
			217	236	206	173	192
			222	228	224	181	195

**Table 6:** Hardness test results of the connections of the #28 mm reinforcing bar with M30, M36, and M42 threaded bars made of steel grade 1.4021 (AISI 420).

Stainless Steel	Joint	Measurement Line	Hardness HV				
			MR_1_	SWC_1_	Welded Zone	SWC_2_	MR_2_
1.4021 (AISI 420)	#28+M30	I	287	374	320	254	199
			280	383	285	249	222
			227	473	249	274	225
		II	222	394	394	224	170
			272	483	424	207	188
			264	689	347	177	192
	#28+M36	I	330	405	311	282	224
			280	446	292	272	198
			232	480	274	250	194
		II	251	429	354	236	198
			236	446	473	214	192
			249	536	324	212	194
	#28+M42	I	254	369	283	214	231
			283	446	249	190	245
			272	560	380	190	276
		II	279	420	336	192	182
			270	429	380	189	192
			236	530	369	203	163

## Data Availability

The original contributions presented in this study are included in the article material. Further inquiries can be directed to the corresponding author.
